# Delivery prediction by quantitative analysis of four steroid metabolites with liquid chromatography tandem mass spectrometry in asymptomatic pregnant women

**DOI:** 10.1080/07853890.2022.2067895

**Published:** 2022-04-25

**Authors:** Lanlan Meng, Shaofei Su, Lin Li, Shengmin Liu, Youran Li, Ying Liu, Yifan Lu, Zhengwen Xu, Lin Liu, Qixin He, Yuanyuan Zheng, Xiaowei Liu, Yuting Cong, Yanhong Zhai, Zhen Zhao, Zheng Cao

**Affiliations:** a Department of Laboratory Medicine, Beijing Obstetrics and Gynecology Hospital, Capital Medical University, Beijing Maternal and Child Health Care Hospital, Beijing, China; b Center of Clinical Mass Spectrometry, Beijing Obstetrics and Gynecology Hospital, Capital Medical University, Beijing Maternal and Child Health Care Hospital, Beijing, China; c Central Laboratory, Beijing Obstetrics and Gynecology Hospital, Capital Medical University, Beijing Maternal and Child Health Care Hospital, Beijing, China; d Health Biotech Co., Ltd, Beijing, China; e Department of Obstetrics, Beijing Obstetrics and Gynecology Hospital, Capital Medical University, Beijing Maternal and Child Health Care Hospital, Beijing, China; f SCIEX, China; g Department of Pathology and Laboratory Medicine, Weill Cornell Medicine, New York, USA

**Keywords:** Delivery prediction, metabolism, LC-MS/MS, steroid, assay validation

## Abstract

**Background:**

Prediction of delivery is important for assessing due dates, providing adequate prenatal care, and suggesting appropriate interventions in preterm and post-term pregnancies. Recent metabolomic findings suggested that the temporal abundance information of metabolome can be used to predict delivery timing with high accuracy in a cohort of healthy women. However, a targeted and quantitative assay is required to further validate the clinical performance and utility of this group of metabolomic candidates in delivery prediction with a larger and independent cohort.

**Method:**

LC-MS/MS quantitative assays were applied to determine the plasma concentrations of four steroid metabolites, including oestriol-16-glucuronide (E3-16-Gluc), 17-alpha-hydroxyprogesterone (17-OHP), tetrahydrodeoxycorticosterone (THDOC), and androstane-3,17-diol (A-3,17-Diol) in asymptomatic women of singleton pregnancies (≥30^th^ gestational weeks). Subsequent statistical analysis was conducted to assess the performance of the above candidates in delivery prediction.

**Result:**

Using LC-MS/MS, four steroids were separated and quantified in 5.5 min. The coefficients of variation (CVs) of the four analytes at the lower limit of quantification ranged from 7.9% to 14.6%, with the *R*^2^ values greater than 0.990 in the calibration curves. Of the 585 recruited pregnant women who ended up with spontaneous delivery, 17.1% and 82.9% of the subjects delivered within and after 7 days since plasma collection, respectively. In the receiver operator curve analysis, the gestational age-adjusted area under the curve of the combined measurements of E3-16-Gluc and 17-OHP was 0.69 (95% CI: 0.60–0.76), with the sensitivity of 87.0% (95% CI: 78.8%–92.9%) and specificity of 60.2% (95% CI: 55.7%–64.6%). Moreover, the positive and the negative predictive values were 28.3%–34.0% and 93.1%–97.4% respectively for this combined panel.

**Conclusion:**

We performed analytical and clinical validation of a quantitation LC-MS/MS panel for the four steroids in the plasma of pregnant women. The steroid metabolites panel of E3-16-Gluc and 17-OHP was potentially useful for predicting delivery within one week in asymptomatic women of singleton pregnancies.

**Key messages**
- A quantitative LC-MS/MS assay for determining the plasma levels of 17-OHP, THDOC, A-3,17-Diol and E3-16-Gluc was developed and validated, in order to evaluate their predictive performance in asymptomatic delivery of singleton pregnancy. The levels of E3-16-Gluc and 17-OHP were found to be significantly elevated at the time of sampling in women that delivered within one week and their combinational testing may be potentially useful in delivery prediction.

## Introduction

Prediction of delivery is important for assessing due dates, providing adequate prenatal care, and suggesting appropriate interventions in preterm and post-term pregnancies [1,2]. Approximately 10% of all pregnancies end up in preterm birth (<37 weeks), which is the leading cause of neonatal morbidity and mortality globally [3,4]. Accurate identification of risk factors for impending preterm delivery is critical to discriminate women with preterm delivery from those who deliver at full term [5]. Furthermore, post-dated pregnancy is frequently associated with complications such as foetal macrosomia, oligohydramnios, increased chance of meconium-stained amniotic fluid and operative intervention [5,6], which could also benefit from reliable delivery prediction.

Current clinical methods of determining gestational age and due date are based on the menstruation period and ultrasound imaging, which are imprecise and depend on accessibility in early pregnancy [7,8]. For the preterm birth prediction in clinical practice, ultrasonic measurement of cervical length (CL) alongside foetal fibronectin (fFN) testing has been shown to improve predictive accuracy and delivery outcome [9,10]. The presence of fFN in cervicovaginal secretions was associated with an increased risk of spontaneous preterm birth (sPTD) in numerous studies [11,12]. However, a meta-analysis demonstrates that the fFN test should not be used as a screening test in asymptomatic pregnant women with or without high risks of sPTD [13]. The development of a simple and accurate diagnostic assay, particularly one implemented in an asymptomatic population, remains an unmet need in clinical practice.

Other laboratory examinations of maternal serum constituents have been added to delivery prediction models [14,15]. Recent work by Liang et al. on untargeted metabolomics in 30 healthy pregnant women showed that a group of 2–3 steroid metabolites, without other inputs from clinical features, might potentially determine the timing of delivery [16]. Importantly, a targeted and quantitative assay is required to further validate the performance and clinical utility of this group of metabolomic candidates in delivery prediction with a larger and independent cohort.

In this study, we developed a liquid chromatography-tandem mass spectrometry (LC-MS/MS) assay to simultaneously quantify the plasma levels of oestriol-16-glucuronide (E3-16-Gluc), 17-alpha-hydroxyprogesterone (17-OHP), tetrahydrodeoxycorticosterone (THDOC), and androstane-3,17-diol (A-3,17-Diol). Furthermore, the clinical performance of those steroid compounds in delivery prediction was evaluated in singleton pregnant women of ≥30 gestational weeks (GWs).

## Materials and methods

### Participants

In this study, the singleton pregnant women that were at 30 GWs and up without any clinical signs of threatened labour, were initially recruited and had their plasma collected upon visiting the Obstetric Department as outpatients at our institute. Those with foetal abnormalities, caesarean section, foetal membrane disruption and induced labour in current pregnancy were excluded. Gestational age was determined in all patients by ultrasound measurement. This study was approved by the Ethics Committee of Beijing Obstetrics and Gynaecology Hospital, Capital Medical University (approval number: 2021-SJ-006-01). The need for informed consent from included individuals was waived by the Ethics Committee as clinical routine blood residual samples were used in this study. The testing results from this study were not used to issue clinical reports and the laboratory data was anonymized before its use.

### Study design

**Figure 1.**
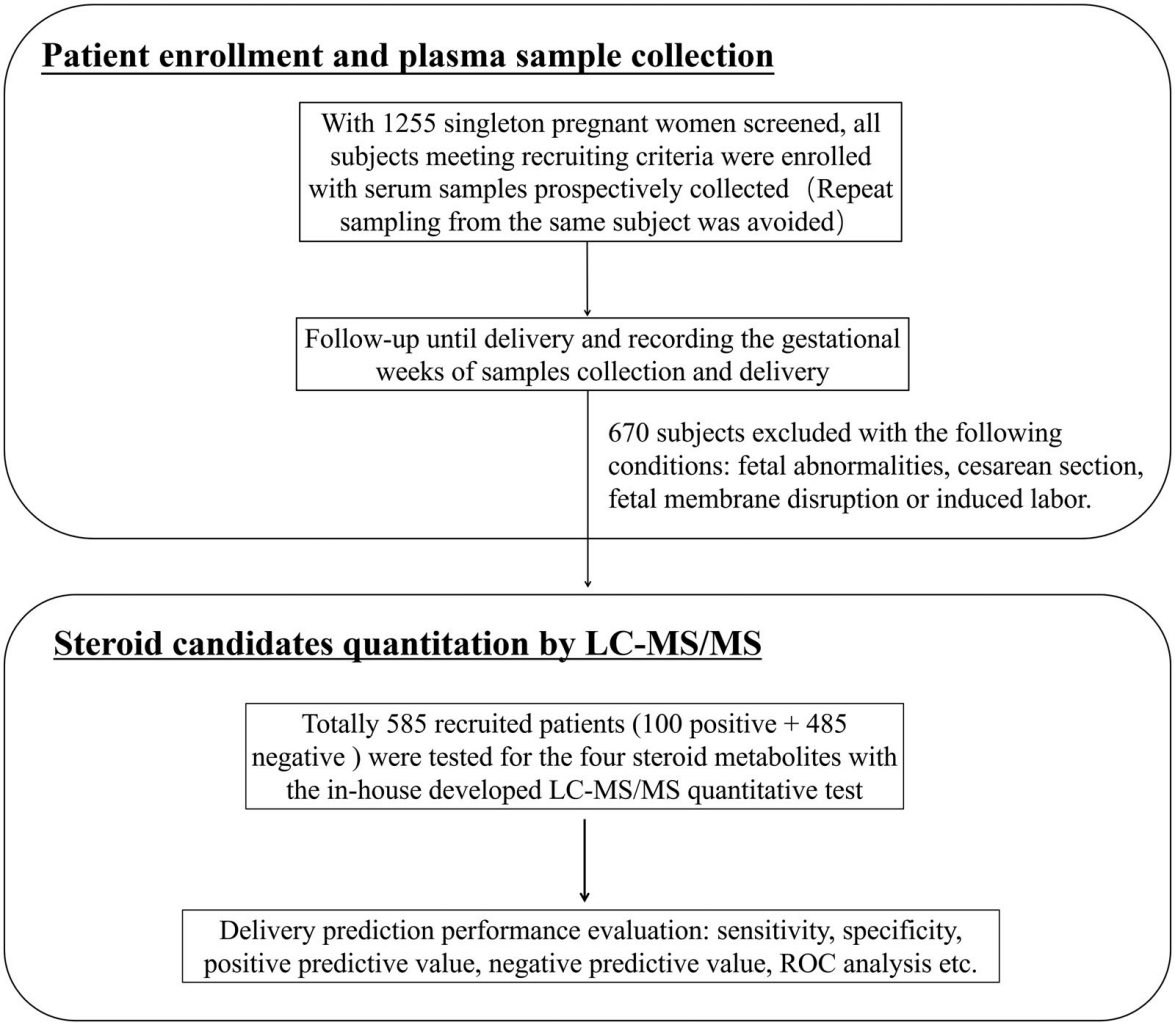
The schematic diagram for patient enrolment and delivery prediction by quantitative analysis of four steroid metabolites with an LC-MS/MS assay.

Asymptomatic pregnant women (≥30^th^ GW) were recruited prospectively (Figure 1). For each participant, 3 ml venous blood was drawn in an EDTA-K_2_ vacuum collection tube, followed by standard centrifugation steps to separate plasma and blood cells. The aliquoted EDTA plasma samples were then stored at −80 °C until tested. Repeat sampling from the same subject was avoided. The basic demographic information of enrolled subjects, and sampling time were extracted from their medical records and analysed retrospectively.

### LC-MS/MS quantitation method

#### Instruments, reagents and chemical standards

The LC-MS/MS analysis was performed using an AB Sciex 5500 mass spectrometer coupled with a Shimadzu Nexera X2 high-performance liquid chromatography (HPLC) system. Methanol (Optima® LC/MS grade), formic acid (LC/MS grade, 98%), and acetonitrile (Optima® LC/MS grade) were purchased from Fisher Scientific; and the LC column used for separation was acquired from Thermo Scientific: Hypersil GOLD 3 μm, 50 × 2.1 mm. The chemical standards including 17-OHP, THDOC, A-3,17-Diol and E3-16-Gluc were purchased from IsoScience (Ambler, Pennsylvania, USA), Cambridge Isotope Laboratories (Cambridge, MA, USA), Zzstandard (Shanghai, China). The internal standards (IS) of 17-OH-P-d8, THDOC-d3, A-3,17-Diol-d3, dehydroepiandrosterone sulphate (DHEAS)-d6 were purchased from Toronto Research Chemicals (Toronto, Canada), Cambridge Isotope Laboratories (Cambridge, MA, USA), IsoScience (Ambler, Pennsylvania, USA).

#### Plasma sample pre-treatment

For the quantitation of E3-16-Gluc and A-3,17-Diol, a liquid-liquid extraction procedure was applied. Briefly, 100 μL collected plasma sample, quality control (QC) or calibrator was mixed with 5 μL of IS working solution followed by addition of 500 μL of methanol/acetonitrile (1:1, v/v) and vortex for 1 min. After centrifugation at 10,000 *g* for 10 min, the supernatant was transferred to a 1.5 mL collection tube to dry under nitrogen at 60 °C for 30 min. Then each extraction was reconstituted with 100 μL of 10% methanol in water, which was ready for LC-MS/MS injection and subsequent analysis. For THDOC and 17-OHP quantitation, essentially the same liquid-liquid extraction steps were followed, except that different precipitation reagents (ethyl acetate/hexane, 1:1 v/v) and reconstitution solution (100% methanol) were used.

#### Instrumental conditions

The liquid chromatographic conditions were set up as follows. The binary mobile phases consisted of 0.1% formic acid (phase A) in water and 100% acetonitrile (phase B) for E3-16-Glu and A-3,17-Diol quantitation. The binary mobile phases used for THDOC and 17-OHP measurement were 0.1% formic acid in water supplemented with 0.2 mg/L lithium chloride (CAS:7447-41-8) (phase A) and 100% methanol (phase B).

The same elution gradient was applied to the two different sets of mobile phases (Supplemental Table 1). The analytes were detected by the mass spectrometer with scheduled multiple reaction monitoring (MRM) in the positive or negative electrospray ionisation mode. The source-specific parameters were as follows: ion spray voltage, 5500 kV or −4500 kV; the temperature was 600 °C; collision gas was set at 6; curtain gas was 20 psi, ion source gas 1 was 55 psi and ion source gas 2 was 55 psi.

#### Method validation

To validate the LC-MS/MS method, its linearity, lower limit of quantification (LLOQ), matrix effect, accuracy (recovery) and precision (intra-assay/inter-assay) were evaluated in accordance with C62-A from the Clinical and Laboratory Standards Institute (CLSI) for bioanalytical method validation [17].

#### Calibrators, quality controls, and internal standard solutions

As the four analytes of interest are internal metabolites of the human body, 5% bovine serum albumin (BSA) in water was used as a matrix blank. For calibrators and QCs, their 20-fold working solutions were prepared with the methanol stock solutions of all the chemical standards at a concentration of 1.0 mg/mL and were stored at −20 °C before use. For daily use, 5 μL of the working solutions and 95 μL of the 5% BSA were mixed to achieve the resulting concentrations of the eight-point calibrators or the QCs (Supplemental Table 2). The internal standard mixture was prepared with methanol and water (1:4) at the following concentrations: 50 ng/mL DHEAS-d6, 50 ng/mL 17-OHP-d8, 50 ng/mL THDOC-d3, and 50 ng/mL A-3,17-Diol-d3.

#### Linearity and LLOQ

The linearity of the assay for each analyte was examined by linear regression analysis [18], in which the calibration curve was created in three separate batches and the average slope, intercept, and correlation coefficient *R*^2^ of the three repeats were reported. The acceptance criterion for a calibration curve was an *R*^2^ of 0.990 or higher. The LLOQs was calculated by analysing the serially diluted QC specimens spiked with IS over 5 days. The LLOQ was defined as the average concentration at which the S/N ratio >10, CV <20% and bias were within ±20% [19].

#### Precision, accuracy and stability

The precision was evaluated by intra-assay precision (*n* = 10 batches) and inter-assay precision (*n* = 25 batches from three separate days) estimation with QC materials. The accuracy was assessed by the recovery studies, in which the recovery of each androgen was calculated at high-, medium-, and low-level QCs by comparing the IS peak area ratio of extracted QC samples to the IS peak area ratio of non-extracted standard solutions at the same concentration [19]. The stability of the analytes in plasma was assessed by evaluating plasma samples kept at 4 and 21 °C (room temperature) for 6 days.

#### Matrix effect, selectivity and carry over

The matrix effect, selectivity and carry-over were assessed according to the CLSI C62-A guideline [17]. Briefly, in the matrix study, sample A was the plasma from 6 healthy pregnant individuals. The sample B was basically QC-L, QC-M, or QC-H prepared in 5% BSA. Then the samples A and B were mixed in different combinations: 100% *A* + 0% B, 20% *A* + 80% B, 50% *A* + 50% B, 80% *A* + 20% B, 0% *A* + 100% B. To be acceptable, the bias between the test results and the theoretical value should be less than 15%. For the selectivity validation, the reagent blank (100% water), matrix blank (5% BSA) and matrix blank (5% BSA) with IS were processed in the same way as patient samples and analysed by the established LC-MS/MS method in the present study, to see if there were any interfering signals from the reagent or matrix. Carryover was assessed by alternating QC-H samples and matrix blank samples five times, to verify the minimal sample carryover. The calculated carry-over in the blank sample should be less than 25% of LLOQ to be acceptable [17,19].

### Statistical analysis

The Kolmogorov–Smirnov/Shapiro–Wilk test was used to determine distribution normality. Chi-square or Fisher’s exact test was used for categorical variables; Student’s *t*-test or Wilcoxon nonparametric test was used for continuous variables. Unadjusted receiver operator curves (ROC) were constructed and the area under the curve (AUC) was calculated to estimate the predictive power of the four steroid candidates for predicting delivery time. Covariate-adjusted ROC curves were obtained by the Nonparametric Bayesian model based on a single-weights-dependent Dirichlet process mixture of normal distributions and the Bayesian bootstrap [20]. The backward selection was used to remove predictors that were not significantly associated with delivery within 7 days (Wald statistic *p* ≥ .05) to obtain the final model. All the statistical analyses were performed using SPSS software (version 25.0, IBM Corp.) or MedCalc (version 19.20). A *p* value less than .05 was considered statistically significant.

## Results

### LC-MS/MS method optimisation

With the LC method described above, the chromatographic separation of the target analytes was achieved within 5.5 min (Supplemental Tables 1 and Supplemental Figures 1, 2). The mass spectrometry instrumentation and conditions, including MRM transitions (including parent and productions of quantifier, qualifier, and internal standard for each analyte), declustering potential (DP), collision energy (CE) and collision cell exit potential (CXP), were optimised for each analyte, with the resulting set-up parameters listed in Supplemental Table 3.

### Assay analytical validation summary

The linearity of the assay was evaluated by linear regression analysis, and the correlation coefficient *R*^2^ values for the four analytes were all greater than 0.990. The linear ranges and the LLOQs of the method were listed as follows: 4–1200 ng/mL, 4.0 ng/mL for E3-16-Gluc and 1–75 ng/mL, 1.0 ng/ml for all the other three analytes. The CVs of the four analytes at their LLOQ levels varied between 7.9% and 14.6% (Table 1), with representative chromatograms shown in Supplemental Figure 3. The intra-assay CV ranges for quality control-low (QC-L), quality control-medium (QC-M) and quality control-high (QC-H) was 8.4%–9.5%, 6.2%–10.7%, and 4.5%–9.2% respectively. The inter-assay CV ranges for QC-L, QC-M and QC-H were 10.8%–11.8%, 5.2%–13.5%, and 4.9%–9.2% respectively. In the accuracy study, the recovery rates ranged from 91. 0% to 118.6% at different QC levels (Table 2). The analytes were found to be stable in plasma for in the recovery range of 80%–120% at least 6 days, when stored at 4 or 21 °C (Table 1).

**Table 1.** Linearity, LLOQ and stability of the four steroid candidates in LC-MS/MS assay.

Analytes	*R* ^2^	Linear range (ng/mL)	Linear regression		LLOQ (ng/mL)	Stability, %	
			Slope (±SD)	Intercept (±SD)		4 °C	21 °C
THDOC	0.997	1–75	0.066 ± 0.005	0.020 ± 0.007	1	98.2	99.5
17-OHP	0.992	1–75	0.627 ± 0.104	0.558 ± 0.057	1	97.5	100.3
A-3,17-Diol	0.992	1–75	0.155 ± 0.022	1.011 ± 0.102	1	101.2	103.4
E3-16-Glu	0.996	4–1200	0.013 ± 0.002	0.087 ± 0.005	4	107.9	110.5

THDOC: tetrahydrodeoxycorticosterone; 17-OHP: 17-alpha-hydroxyprogesterone; A-3,17-Diol: androstane-3,17-diol; E3-16-Glu: oestriol-16-glucuronide; SD: standard deviation; stability: recovery rates for plasma samples stored at 4 or 21 °C for 6 days.

**Table 2.** Recoveries and imprecisions of the four steroid candidates in LC-MS/MS assay.

	E-16-Gluc	THDOC	A-3,17-Diol	17-OHP
Recovery, %				
QC-L	97.5	92.6	101.0	118.6
QC-M	91.2	109.4	92.4	117.0
QC-H	107.9	91.0	88.3	102.0
Intra-assay CV, % (*n* = 10)				
QC-L	8.4	9.1	8.6	9.5
QC-M	6.9	6.9	10.7	6.2
QC-H	9.2	7.8	7.2	4.5
Inter-assay CV, % (*n* = 25)				
QC-L	11.8	11.1	10.8	10.9
QC-M	12.2	5.2	10	13.5
QC-H	4.9	8.4	9.2	5.2

QC-L: low level quality control; QC-M: medium level quality control, QC-H: high level quality control.

No essential matrix effect was observed in the various mixtures of 5% BSA and QCs, with the deviation between the test results and the theoretical value less than 15%. Similarly, there was no detectable selectivity interference (Supplemental Figure 4) seen in our method validation study. Moreover, the carry-over for all the four analytes was acceptable (<25% LLOQ in the matrix blank sample) (data not shown).

### Clinical validation: Delivery prediction by the steroid candidates quantified by LC-MS/MS

From October 2020 to January 2021, 1500 singleton women were screened and had their plasma samples collected upon initial assessment. After a retrospective review of the patients’ medical records, 585 participants with spontaneous delivery and meeting other recruiting criteria were included for subsequent LC-MS/MS analysis (Figure 1).

The demographic and obstetrical characteristics of the participants were listed in Table 3. Of the recruited participants, 100 (17.1%) women delivered within 7 days after plasma collection (the positive group), while 485 (82.9%) women delivered after 7 days since sampling (the negative group). The median gestational age at the time of sampling was 34 gestational weeks (GWs) (32–37 GWs), and the median gestational age at delivery was 39 GWs (38–40 GWs). The plasma levels of the four steroid candidates were included in Table 3.

**Table 3.** Demographic data of enrolled subjects and plasma levels of the four steroid candidates.

	Positive group* (*n* = 100, 17.1%)	Negative group* (*n* = 485, 82.9%)	Unadjusted *p*	GA-adjusted *p*
Demographic data				
Age	32 (29, 34)	31 (29, 33)	0.6	–
GA of plasma collection	38 (37,3 9)	33 (32, 36)	<0.001	–
GA of spontaneous delivery	39 (38, 40)	39 (39, 40)	0.2	–
Plasma levels (ng/ml)				
E3-16-Glu	250.5 (174.0, 362.0)	133.0 (87.8, 204.0)	<0.001	<0.001
THDOC	0.7 (0.5, 1.2)	0.8 (0.5, 1.1)	0.67	0.71
A-3,17-Diol	1.7 (0.4, 4.2)	1.8 (0.5, 4.6)	0.76	0.62
17-OHP	7.9 (5.5, 11.6)	5.3 (3.4, 8.0)	<0.001	0.02

Positive group: the patients delivering within 7 days of sample collection; negative group: the patients delivering 7 days after sample collection; GA: gestational age. *Data presented as median (25th, 75th) percentile.

**Figure 2.**
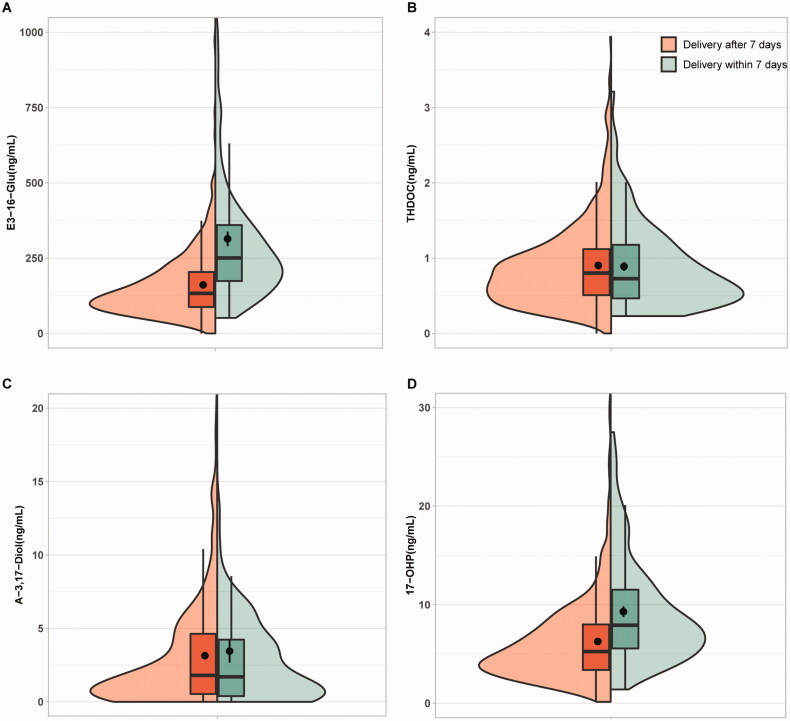
Box plots representing the plasma levels of the four steroid candidates in positive and negative groups. (A) E3-16-Glu, oestriol-16-glucuronide; (B) THDOC, tetrahydrodeoxycorticosterone; (C) A-3,17-Diol, androstane-3,17-diol；(D) 17-OHP, 17-alpha-hydroxyprogesterone. *Indicates *p* < .001.

As shown in Supplemental Figure 2, all the plasma levels of the four steroid metabolites measured by our LC-MS/MS assay were gradually elevated as the gestational age (GA) went up, which was similar to the previous observation [16]. The concentrations of THDOC and A-3,17-Diol displayed no significant difference between the positive and negative groups. By contrast, the plasma levels of E3-16-Gluc and 17-OHP were significantly higher in the positive group than those in the negative group with or without adjustment (*p* < .05) (Figure 2, Table 3).

**Figure 3.**
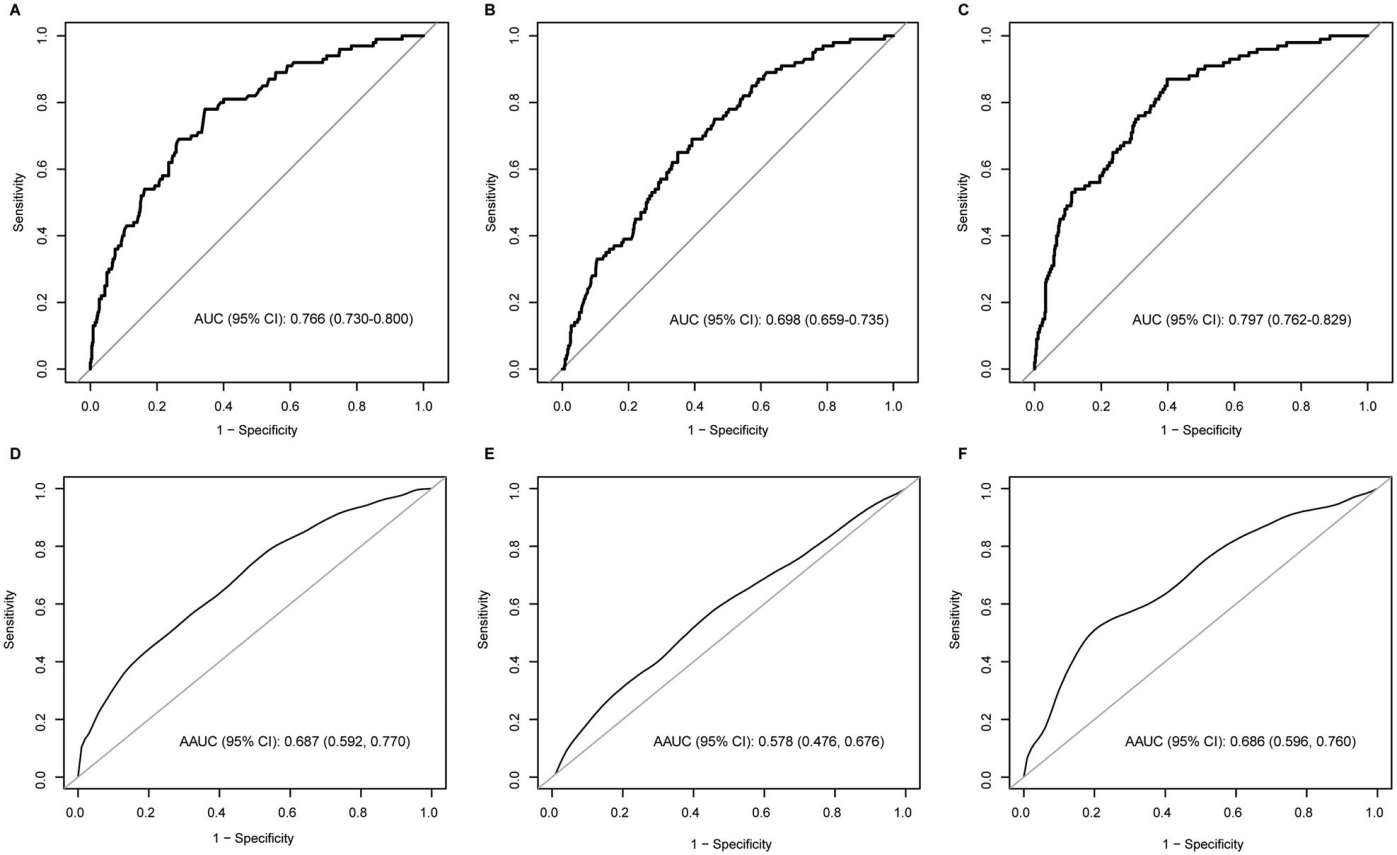
Performance assessment of the steroid candidates E3-16-Gluc and 17-OHP in delivery prediction by ROC analysis with or without gestational age adjustment. (A–C): ROC curves for E3-16-Gluc, 17-OHP, and combined testing (E3-16-Gluc and 17-OHP); (D–F): gestational age-adjusted ROC curves for E3-16-Gluc, 17-OHP, and combined testing (E3-16-Gluc and 17-OHP) respectively. AUC: area under the curve; AAUC: gestational age-adjusted AUC.

In the evaluation of the performance of the four steroid candidates in delivery prediction, the crude and GA-adjusted AUC and 95% confidence intervals are presented in Table 4. With the unadjusted-ROC analyses, the AUCs of E3-16-Gluc, THDOC, A-3,17-Diol and 17-OHP were 0.77 (95% confidence interval (CI): 0.73–0.80), 0.51 (95% CI: 0.47–0.56), 0.51 (95% CI: 0.47–0.55), and 0.70 (95% CI: 0.66–0.74), respectively (Figure 3, Table 4). After GA adjustment, the AUCs of E3-16-Gluc and 17-OHP were 0.69 (95% CI: 0.59–0.77) and 0.58 (95% CI: 0.48–0.68) respectively; the AUC of the combined analytes (E3-16-Gluc and 17-OHP) was 0.69 (95% CI: 0.60–0.76), which was not significantly superior to that of E3-16-Gluc (*p* > .05) (Figure 3, Table 4).

**Table 4.** Predictive performance of the four steroid candidates in asymptomatic pregnant women delivery.

Predictive performance	E3-16-Glu	THDOC	A-3,17-Diol	17-OHP	Combination
Sensitivity, 95% CI (%)	78.0 (68.6–85.7)	41.0 (31.3–51.3)	55.0 (44.7–65.0)	65.0 (54.8–74.3)	87.0 (78.8–92.9)
Specificity, 95% CI (%)	65.6 (61.2–69.8)	67.2 (62.8–71.4)	38.8 (34.4–43.3)	65.2 (60.7–69.4)	60.2 (55.7–64.6)
PPV, 95% CI (%)	31.8 (28.4–35.4)	20.5 (16.5–25.2)	15.6 (13.3–18.3)	27.8 (24.2–31.7)	31.1 (28.3–34.0)
NPV, 95% CI (%)	93.5 (90.9–95.5)	84.7 (82.3–86.8)	80.7 (76.6–84.2)	90.0 (87.3–92.2)	95.7 (93.1–97.4)
Unadjusted AUC, 95% CI (%)	0.77 (0.73–0.80)	0.51 (0.47–0.56)*	0.51 (0.47–0.55)*	0.70 (0.66–0.74)*	0.80 (0.76–0.83)
GA-adjusted AUC, 95% CI (%)	0.69 (0.59–0.77)	ND	ND	0.58 (0.48–0.68)*	0.69 (0.60–0.76)

Combination: combination detection of E3-16-Glu and 17-OHP; PPV: positive predictive value; NPV: negative predictive value; GA: gestational age; CI: confidence interval. *Indicates *p* < .05, when compared with the AUC of the combination detection of E3-16-Glu and 17-OHP in ROC analysis.

As shown in Table 4, with the Youden Index (sum of sensitivity and specificity minus one) determined from the ROC analyses, the sensitivities for E3-16-Gluc, THDOC, A-3,17-Diol, 17-OHP and E3-16-Gluc + 17-OHP were 78.0% (95% CI: 68.6–85.7%), 41.0% (95% CI: 31.3–51.3%), 55.0% (95% CI: 44.7–65.0%), 65.0% (95% CI: 54.8–74.3%) and 87.0% (95%CI: 78.8–92.9%) respectively; the specificities for the above analytes or combined two-marker panel were 65.6% (95% CI: 61.2–69.8%), 67.2% (95% CI: 62.8–71.4%), 38.8% (95% CI: 34.4–43.3%), 65.2% (95% CI: 60.7–69.4%) and 60.2% (95% CI: 55.7–64.6%) respectively. The positive predictive values (PPVs) were ranged between 28.3% and 34.0%, and the negative predictive values (NPVs) were between 93.1% and 97.4% (Table 4), demonstrating a far better performance in ruling out than ruling in spontaneous delivery within 7 days of testing.

## Discussion

We developed and performed analytical and clinical validation of a quantitative LC-MS/MS assay to determine the plasma levels of 17-OHP, THDOC, A-3,17-Diol and E3-16-Gluc in pregnant women. The combined E3-16-Gluc and 17-OHP panel showed clinical values to predict delivery within one week in asymptomatic pregnant women after 30 GWs.

Plasma concentrations of E3-16-Gluc and17-OHP were both found significantly elevated in the patient group who delivered within 7 days of sample collection. The relevance between the two steroid metabolites and delivery has been briefly investigated in previous studies. For instance, the relative increase of E3-16-Gluc during pregnancy was reported by Beling et al. in 1963 [21]. Interestingly, the measurement of free oestriol has been suggested for delivery prediction as it was considered a good index of placental aromatisation and the onset of labour process [22–24]. In addition, in singleton pregnancies with high-risk conditions such as sonographic short cervix or a history of previous preterm birth, the measurement of vaginal progesterone or 17-hydroxyprogesterone caproate was found to be able to reduce the rate of preterm birth [25–27].

With the cut-off values determined by the ROC analysis, the NPV of the combined measurements of E3-16-Gluc and17-OHP was as high as 95.7%. The ability to accurately rule out spontaneous delivery within a relatively short window (i.e. 7 days) is likely to improve clinical decisions with regard to hospitalisation versus outpatient monitoring and the intensity of outpatient monitoring. It was estimated that around 50% of the hospitalised pregnant women with preterm birth symptoms eventually ended up giving birth at full term [28,29]. In addition, it would also be clinically valuable to prevent overdiagnosis and treatment of preterm birth, with the help of reliable markers for delivery prediction. Similar to the fFN assessment which is designed for premature delivery prediction in asymptomatic high-risk women or symptomatic women of threatened preterm labour, the two-marker panel in the present study showed a relative low PPV of 31.3% in asymptomatic women, implying limited power in predicting the occurrence of spontaneous delivery within a short observation window of 7 days [30,31]. Nevertheless, the two steroid molecules, E3-16-Gluc and 17-OHP, reflected the pathophysiological metabolism changes that might cause the onset of delivery [32] and therefore its application in delivery prediction can be expanded to asymptomatic or low-risk women which represent a larger clinical need.

The value of “hormone profile” measurement in preterm prediction has been recommended in other relevant studies using serum or saliva samples, in which simultaneous quantitation of multiple hormones was superior to a single measurement [33–35]. In our study, the predictive performance of the combined testing of the E3-16-Gluc and 17-OHP was comparable to that of E3-16-Gluc alone, although the overall prediction model still needs to be further optimised in the larger population.

There are limitations to this study. As asymptomatic pregnant women from outpatient clinics were recruited, the majority of the subjects were delivered at full term and only a few participants experienced preterm birth (*n* = 8). The predictive performance of the steroid candidates in preterm delivery needs to be validated separately using independent cohorts targeting asymptomatic high-risk women and/or symptomatic women in threatened preterm labour. Secondly, it is a single-centre study, a multi-centre study involving different geographic areas can be more representative in a future validation study.

## Conclusion

In summary, using the in-house developed and validated quantitative LC-MS/MS method, for the first time we showed that the combined measurements of E3-16-Gluc and 17-OHP in plasma could be potentially useful for predicting delivery within one week in asymptomatic women of singleton pregnancies.

## Supplementary Material

Supplemental Material

## Data Availability

The authors confirm that the data supporting the findings of this study are available within the article and its Supplemental materials.
