# Supplementary material for: Delivery prediction by quantitative analysis of four steroid metabolites with liquid chromatography tandem mass spectrometry in asymptomatic pregnant women
Source: Ann Med. 2022 Apr 25;54(1):1150–9. doi: 10.1080/07853890.2022.2067895 (PMC9045778; doi:10.1080/07853890.2022.2067895)
Supplement: Supplemental Material [file IANN_A_2067895_SM4307.zip › Supplemental file/Supplementary Tables Delivery prediction 0401.docx]

**Supplemental Table 1** The elution gradient and conditions used in the LC-MS/MS assay

|  |  |  |  |  |
| --- | --- | --- | --- | --- |
| Step | Time, min | Flow rate, mL/min | Phase A (%) | Phase B (%) |
| 1 | 0 | 0.5 | 80 | 20 |
| 2 | 0.5 | 0.5 | 80 | 20 |
| 3 | 2 | 0.5 | 20 | 80 |
| 4 | 4.8 | 0.5 | 20 | 80 |
| 5 | 5 | 0.5 | 80 | 20 |
| 6 | 5.5 | 0.5 | 80 | 20 |
|  |  |  |  |  |

**Supplemental Table 2** The concentrations of quality controls and calibrators

|  |  |  |  |  |
| --- | --- | --- | --- | --- |
|  | E-16-Gluc | THDOC | A-3,17-Diol | 17-OHP |
| QC concentration (ng/ml) |  |  |  |  |
| QC-L | 20 | 2.5 | 2.5 | 2.5 |
| QC-M | 250 | 12.5 | 12.5 | 12.5 |
| QC-H | 1000 | 50 | 50 | 50 |
| Eight-point calibrators (ng/ml) | |  |  |  |
| Calibrator-1 | 4.0 | 1.0 | 1.0 | 1.0 |
| Calibrator-2 | 20 | 2.5 | 2.5 | 2.5 |
| Calibrator-3 | 50 | 5.0 | 5.0 | 5.0 |
| Calibrator-4 | 100 | 10 | 10 | 10 |
| Calibrator-5 | 200 | 20 | 20 | 20 |
| Calibrator-6 | 500 | 25 | 25 | 25 |
| Calibrator-7 | 1000 | 50 | 50 | 50 |
| Calibrator-8 | 1200 | 75 | 75 | 75 |
| QC-L: low level quality control; QC-M: medium level quality control, QC-H: high level quality control. | | | | |

**Supplemental Table 3** The mass spectrometer parameters used in the LC-MS/MS assay

|  |  |  |  |  |  |  |  |
| --- | --- | --- | --- | --- | --- | --- | --- |
| Analytes | ESI | Q1, m/z | Product ions | Q3, m/z | DP, V | CE, V | CXP, V |
| THDOC | + | 341.3 | Quantifier | 93.1 | 110 | 50 | 9 |
|  |  |  | Qualifier | 107 | 80 | 50 | 9 |
| THDOC-D3 |  | 338.1 | IS | 320.1 | 110 | 10 | 9 |
| 17-OH-P | + | 331.4 | Quantifier | 109.2 | 130 | 30 | 9 |
|  |  |  | Qualifier | 97.1 | 110 | 30 | 9 |
| 17-OHP-D8 |  | 339.3 | IS | 112.2 | 140 | 30 | 12 |
| A-3,17-Diol | + | 275 | Quantifier | 257.2 | 110 | 20 | 9 |
|  |  |  | Qualifier | 149.1 | 110 | 20 | 6 |
| A-3,17-Diol-D3 |  | 278.2 | IS | 260 | 110 | 20 | 6 |
| E3-16-Glu | - | 463.2 | Quantifier | 112.8 | -130 | -40 | -18 |
|  |  |  | Qualifier | 287.1 | -140 | -40 | -15 |
| DHEAS-D6 |  | 373.0 | IS | 98 | -50 | -50 | -10 |
| THDOC: tetrahydrodeoxycorticosterone; 17-OHP: 17-alpha-hydroxyprogesterone; A-3,17-Diol: androstane-3,17-diol; E3-16-Glu: estriol-16-glucuronide; DHEAS: dehydroepiandrosterone sulfate; IS: internal standard; DP: declustering potential; CE: collision energy; CXP: collision cell exit potential. | | | | | | | |
