# Supplementary figures and images for: Delivery prediction by quantitative analysis of four steroid metabolites with liquid chromatography tandem mass spectrometry in asymptomatic pregnant women
Source: Ann Med. 2022 Apr 25;54(1):1150–9. doi: 10.1080/07853890.2022.2067895 (PMC9045778; doi:10.1080/07853890.2022.2067895)

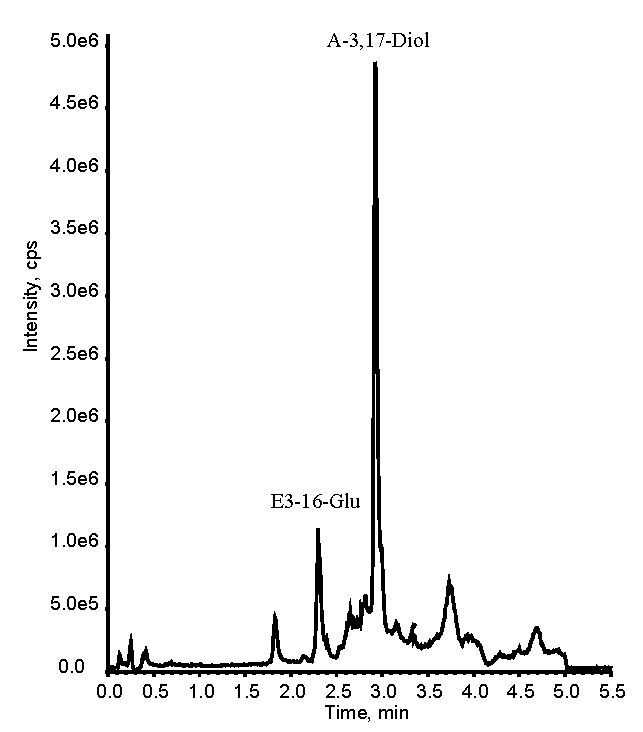

Supplement: Supplemental Material [file IANN_A_2067895_SM4307.zip › Supplemental file/Suppl Fig 1A E316Gluc and A317Diol.tif]

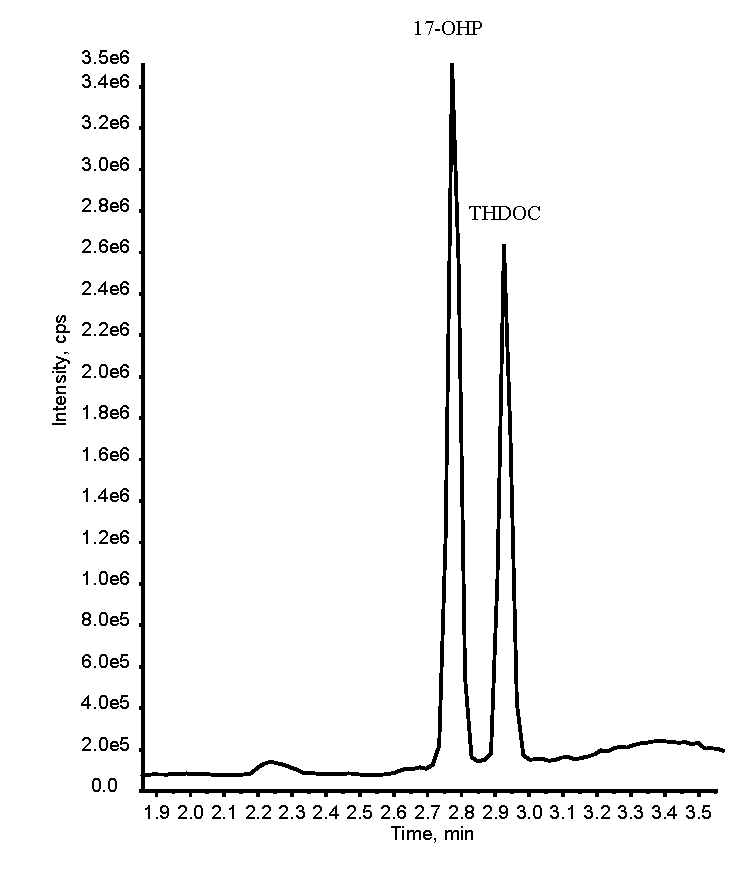

Supplement: Supplemental Material [file IANN_A_2067895_SM4307.zip › Supplemental file/Suppl Fig 1B THDOC and 17OHP.tiff]

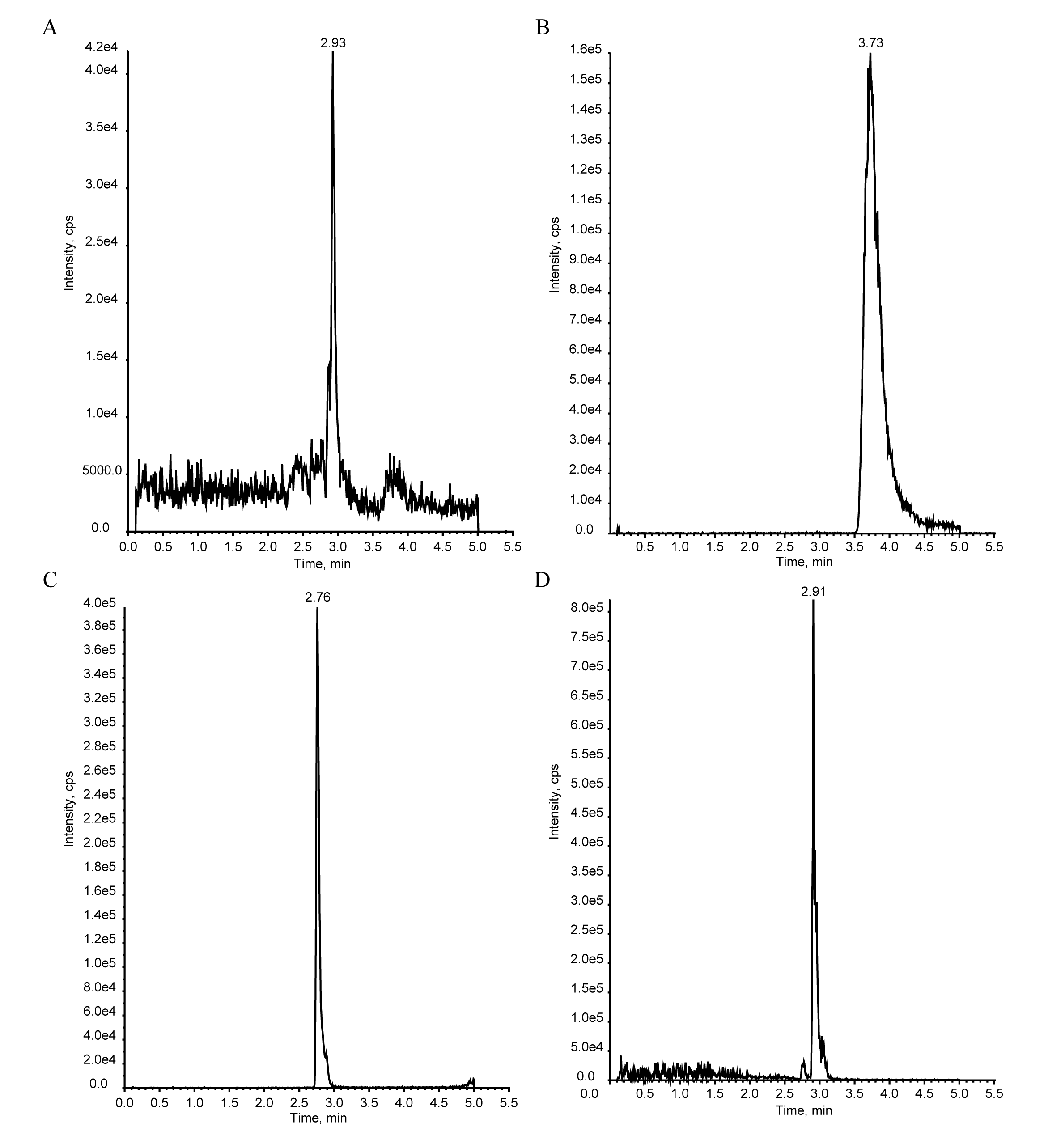

Supplement: Supplemental Material [file IANN_A_2067895_SM4307.zip › Supplemental file/Suppl Fig 2 Internal standards.tif]

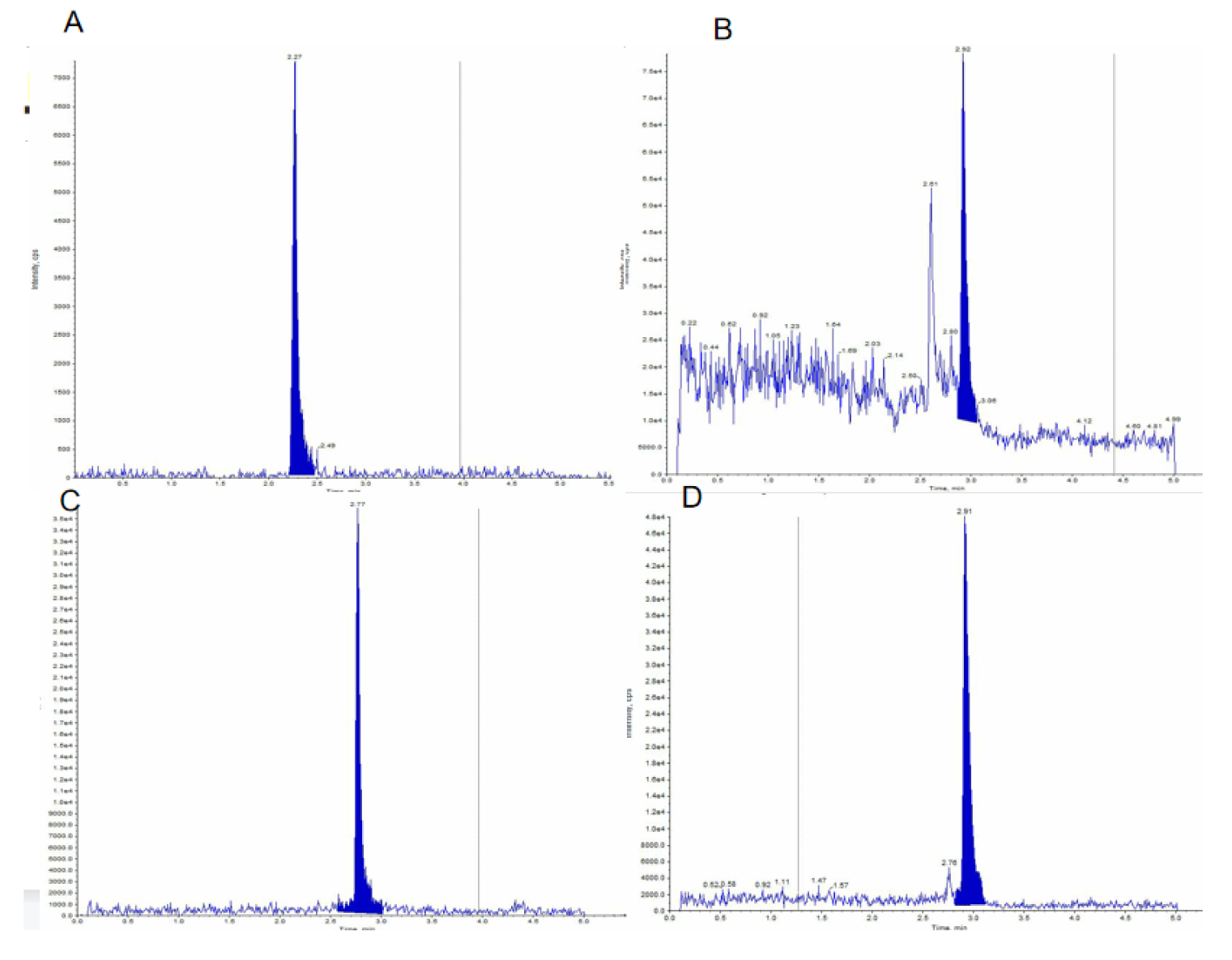

Supplement: Supplemental Material [file IANN_A_2067895_SM4307.zip › Supplemental file/Suppl Fig 3 LLOQ.tif]

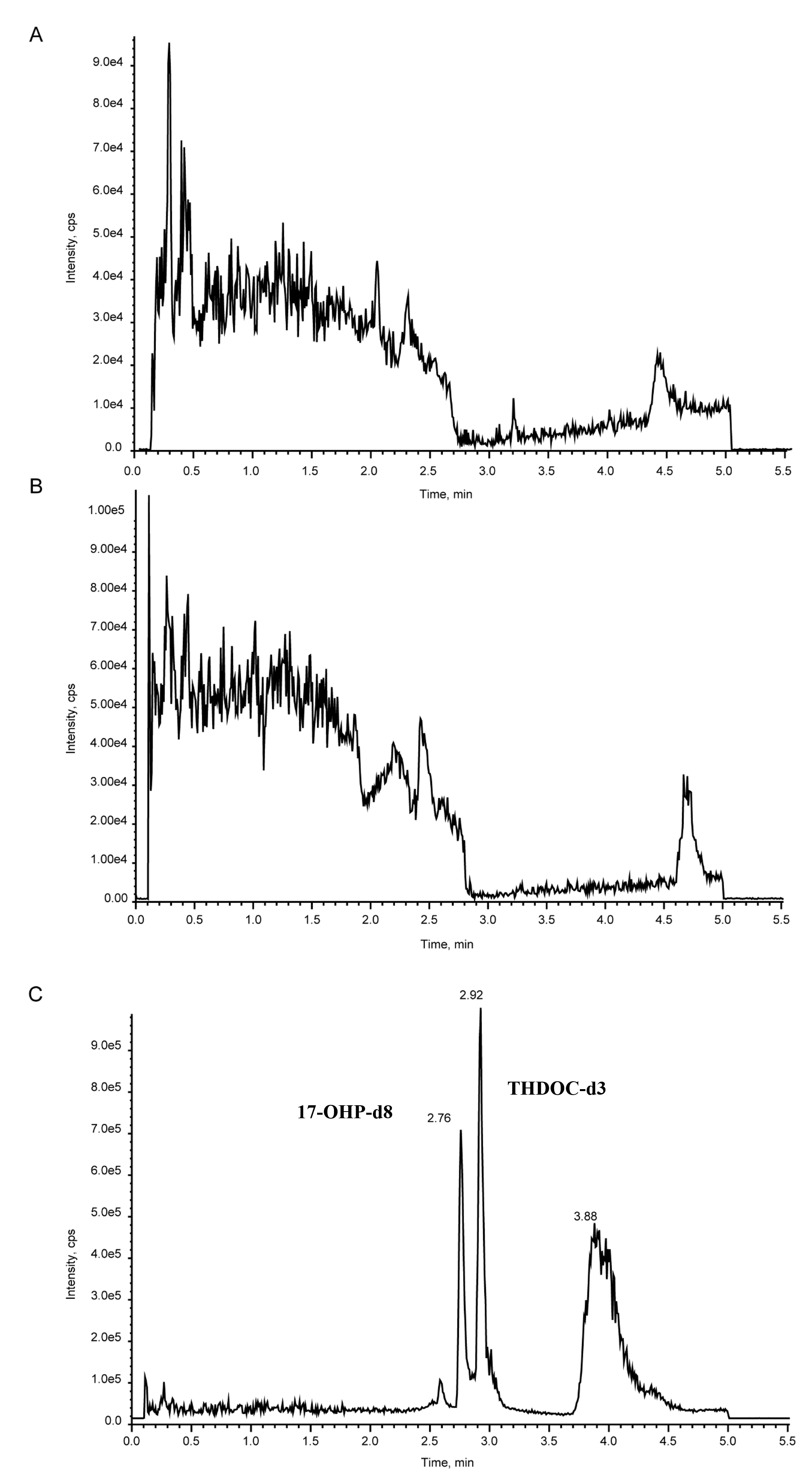

Supplement: Supplemental Material [file IANN_A_2067895_SM4307.zip › Supplemental file/Suppl Fig 4 Selectivity.tif]

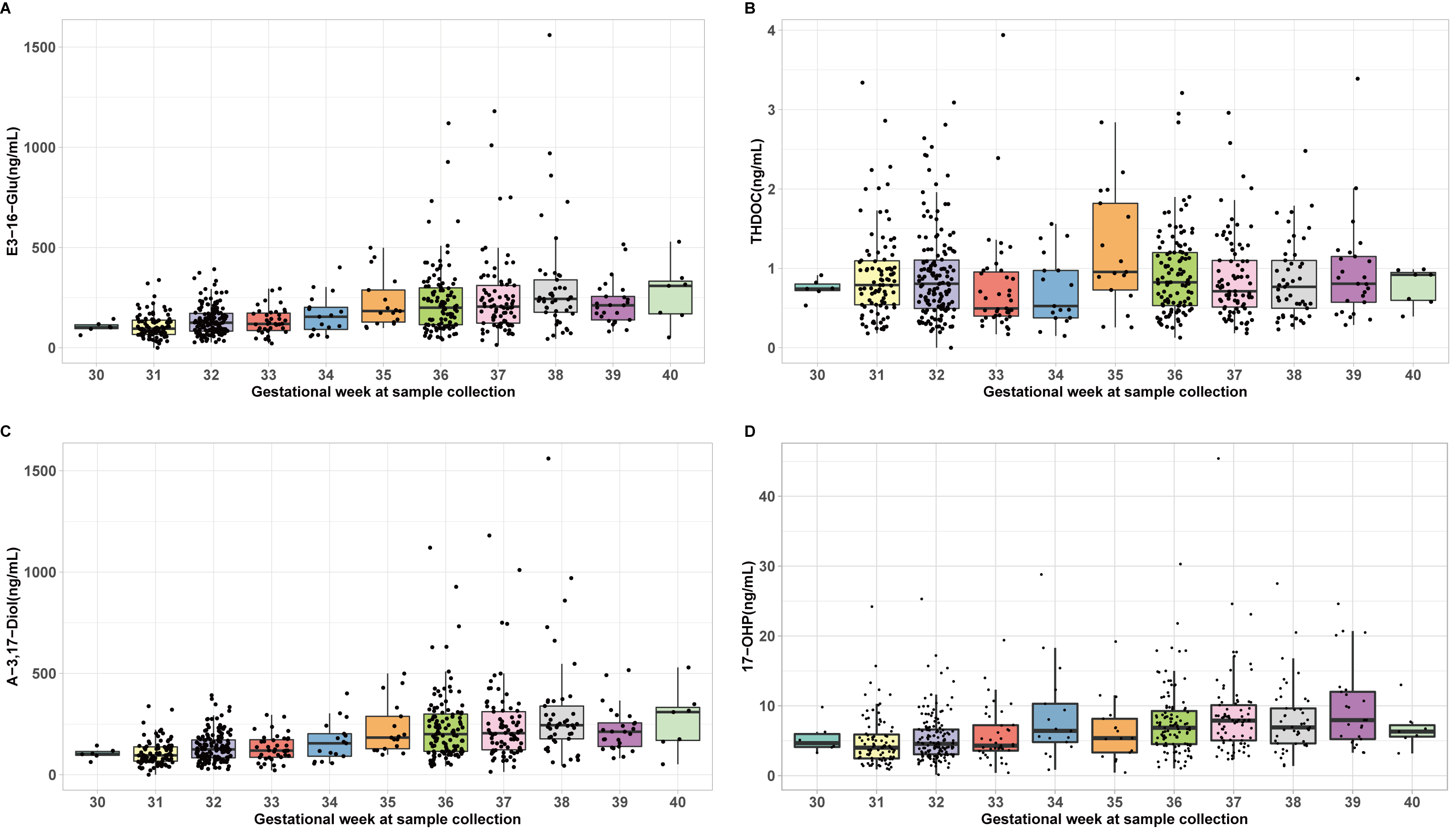

Supplement: Supplemental Material [file IANN_A_2067895_SM4307.zip › Supplemental file/Suppl Fig 5 Change over GA.tif]
